# Impact of COVID‐19 on schooling in rural Zimbabwe

**DOI:** 10.1111/cch.12955

**Published:** 2022-10-12

**Authors:** Joe D. Piper, Clever Mazhanga, Dzivaidzo Chidhanguro, Andrew J. Prendergast, SHINE Trial Team

**Affiliations:** ^1^ Zvitambo Institute for Maternal and Child Health Harare Zimbabwe; ^2^ Blizard Institute Queen Mary University of London London UK

**Keywords:** child health, COVID‐19, education, inequity, lockdown, rural, sub‐Saharan Africa, Zimbabwe

The indirect effects of COVID‐19 on children's education, health, and well‐being due to school closures have raised concerns, particularly in low‐ and middle‐income countries (LMICs) (Armitage & Nellums, 2020; Coker et al., 2021; Zar, Dawa, Fischer, & Castro‐Rodriguez, 2020). In LMICs, children may not have equitable access to distance learning; indeed, the poorest children are likely to suffer most, including reduced nutritional intake without access to school meals (Coker et al., 2021). As lockdown cycles continue globally, alternative methods of supporting children's education become ever more urgent (Coker et al., 2021). Although some opportunities for learning have been conceived, such as radio programmes, there has been little direct measurement of household‐level education during lockdowns in rural sub‐Saharan Africa.

Here, we report a survey of 80 subsistence farming households in rural Zimbabwe during lockdown. Primary caregivers of 7‐year‐old children were interviewed following written informed consent between September and December 2020, as part of a study on school‐age growth and cognition. All caregivers reported children missing school due to COVID‐19. Towards the end of this study, schools began to reopen: 15 children were enrolled in school, but only 3 were actually attending school. Among children not attending school, only half were receiving home schooling, defined by asking the caregiver whether the child was taught lessons at home in subjects they would normally learn at school. Home schooling mostly relied on parents or local adults (Table 1). Parents providing home schooling had 1.7 more years of education (95%CI 0.3, 3.1, *p* = 0.02) than those who did not. About 15% (*N* = 12) households reported receiving support in teaching, 9% (*N* = 7) from schools. Only 6% (*N* = 5) households received additional educational support using radio, phone or online resources, despite 74% having access to a radio and 99% access to a phone.

**TABLE 1:** Access to home schooling during lockdown for 80 children aged 7 years in rural Zimbabwe

Total *N* = 80 (39 girls, 41 boys)	
Median age 7 years 7 months (IQR 7 years 5 months, 7 years 10 months)	
Enrolled at school	15/80
Child attending school	3/80 (4%)
No schooling	44/80 (55%)
Home schooling:	33/80 (41%)
By parents	15/80 (19%)
By another adult	13/80 (16%)
By teacher	1/80 (1%)
By >1 person	4/80 (5%)
Receiving support with home schooling	12/80 (15%)
Learning support from school	7/80 (9%) (three attending school)
Alternative schooling support:	5/80 (6%)
By radio	2/80 (3%)
By phone	2/80 (3%)
By online learning	1/80 (1%)
Household asset ownership:	
TV	36/80 (45%)
Radio	59/80 (74%)
Phone	79/80 (99%)
Solar panel	62/80 (78%)
Mains electricity	9/80 (11%)

From January to March 2021, school reopening in Zimbabwe was suspended again due to the re‐emergence of COVID‐19. The long‐term impact of school closures is profound: the Organisation for Economic Co‐operation and Development (OECD) predicted that a 4‐month learning disruption would conservatively cost nations 1.5% GDP, equivalent to US$504 billion for South Africa alone (Hanushek & Woessman, 2020). In LMICs, there is urgent need for an equity‐based approach to mitigate the loss of schooling, which risks further driving inequity, and creating a lifelong impact on health, well‐being and human capital (The Lancet Public Health, 2020).

The widespread availability of radio, solar panels and mobile phones highlighted in this survey shows the opportunities for creative solutions to deliver remote education. A coordinated approach to facilitating educational support, targeted across the digital divide and prioritizing all children, is urgently required to mitigate the intergenerational human and economic cost that countries will otherwise incur.

## FUNDING INFORMATION

This work was funded by Wellcome Trust (grant number 220671/Z/20/Z and 108,065/Z/15/Z), National Institutes of Health (NIH, grant number R61HD103101) and research grant from the Thrasher Research Fund.

## CONFLICT OF INTEREST

There are no conflicts of interest from any authors.

## ETHICS STATEMENT

Ethical approval was provided by the Medical Research Council of Zimbabwe (MRCZ).

## AUTHOR CONTRIBUTION

JDP conceptualized the project, performed data curation, methodology, funding acquisition, methodology, formal analysis and writing. CM undertook data curation and contributed to methodology, project administration and supervision. DC undertook data curation and contributed to methodology, project administration and supervision. AJP contributed to conceptualisation, methodology, resources, supervision, visualization and writing‐review and editing.
